# Transapical Mitral TEER and Aortic Regurgitation TAVR With Dedicated Device in a Patient With Extreme Scoliosis

**DOI:** 10.1016/j.jaccas.2026.109582

**Published:** 2026-09-30

**Authors:** Dominador V. del Castillo, Bin Wang, Yan Wang

**Affiliations:** Department of Cardiology, Cardiovascular Hospital, Xiamen University, Xiamen, China; b Division of Structural Heart Disease, Xiamen Cardiovascular Hospital Xiamen University, Xiamen, China; c Division of Structural Heart Disease, Xiamen Cardiovascular Hospital Xiamen University, Xiamen, China

**Keywords:** heart team approach, multivalvular transcatheter valve intervention, one-stage transcatheter intervention, severe scoliosis, structural heart disease

## Abstract

**Background:**

Concomitant aortic and mitral regurgitation (CAMR) is a rare valvulopathy, seen in patients with scoliosis, and rarely involves aortic regurgitation with concomitant primary mitral regurgitation associated with poor prognosis when left untreated. Current guidelines lack robust evidence to guide the management of CAMR in complex anatomies such as extreme scoliosis.

**Case Summary:**

We report the first case of a one-stage transcatheter intervention for CAMR in a 62-year-old man with extreme scoliosis. The patient underwent one-stage transapical mitral transcatheter edge-to-edge repair (mTEER) and on-label transcatheter aortic valve replacement to reduce surgical risk in high-risk patients. Despite significant anatomical challenges, the procedure was completed successfully without complications.

**Conclusion and Take-Home Messages:**

CAMR in patients with severe scoliosis poses high operative risk, but a one-stage transapical approach using dedicated mTEER and transcatheter aortic valve replacement is a feasible, effective strategy. An mTEER-first strategy enables hemodynamic stabilization and precise leaflet grasping. This case underscores the value of individualized transcatheter strategies to achieve safe and effective outcomes in complex valvular disease.

## Graphical Abstract

**Figure:**
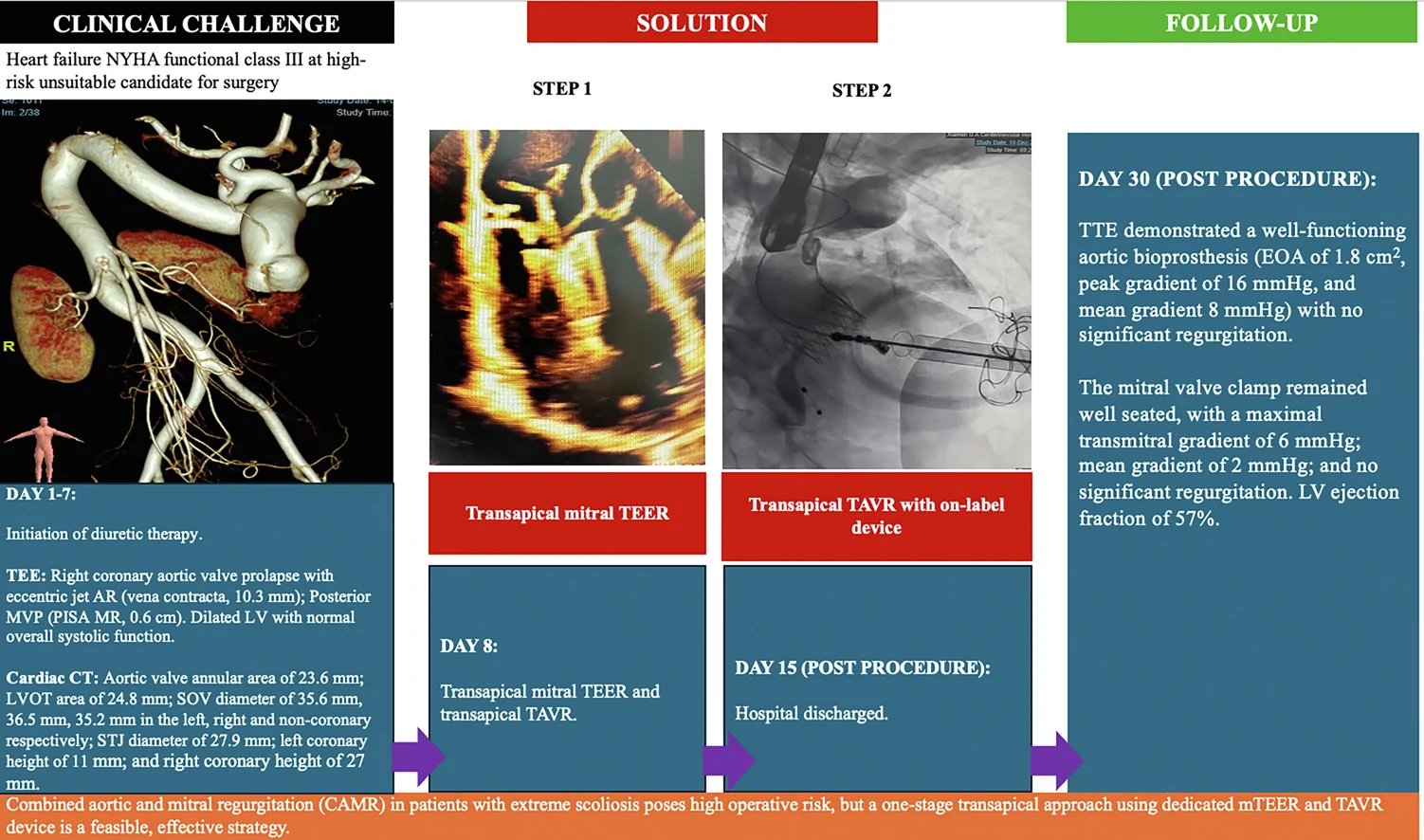

## History of Presentation

This case report presents a 62-year-old man with worsening exertional dyspnea over the past 3 months to NYHA functional class III associated with bipedal edema and orthopnea despite receiving optimal medical management. Transthoracic echocardiography revealed severe aortic regurgitation (AR) and mitral regurgitation (MR), with left ventricular ejection fraction of 53% and a left ventricular end-diastolic diameter of 58 mm prompting admission for the management of hemodynamically significant concomitant aortic and mitral regurgitation (CAMR).

**Take-Home Messages**

• Combined aortic and mitral regurgitation in patients with severe scoliosis poses high operative risk, but a one-stage transapical approach using a dedicated mitral transcatheter edge-to-edge repair and transcatheter aortic valve replacement device is a feasible, effective strategy.
• An mTEER-first strategy enables hemodynamic stabilization and precise leaflet grasping.
• This case underscores the value of individualized transcatheter strategies to achieve safe and effective outcomes in complex valvular disease.

The patient has severe scoliosis deformity with Cobb's angle of 120°, ambulating independently with a mildly uneven gait (Figure 1). On auscultation, a grade 4/6 systolic murmur along the cardiac apex and a grade 3/6 early diastolic decrescendo murmur over the left sternal border were observed. A wide pulse pressure (80-90 mm Hg) with bounding peripheral pulses and bilateral pitting edema of the lower extremities were evident.

**Figure 1:**
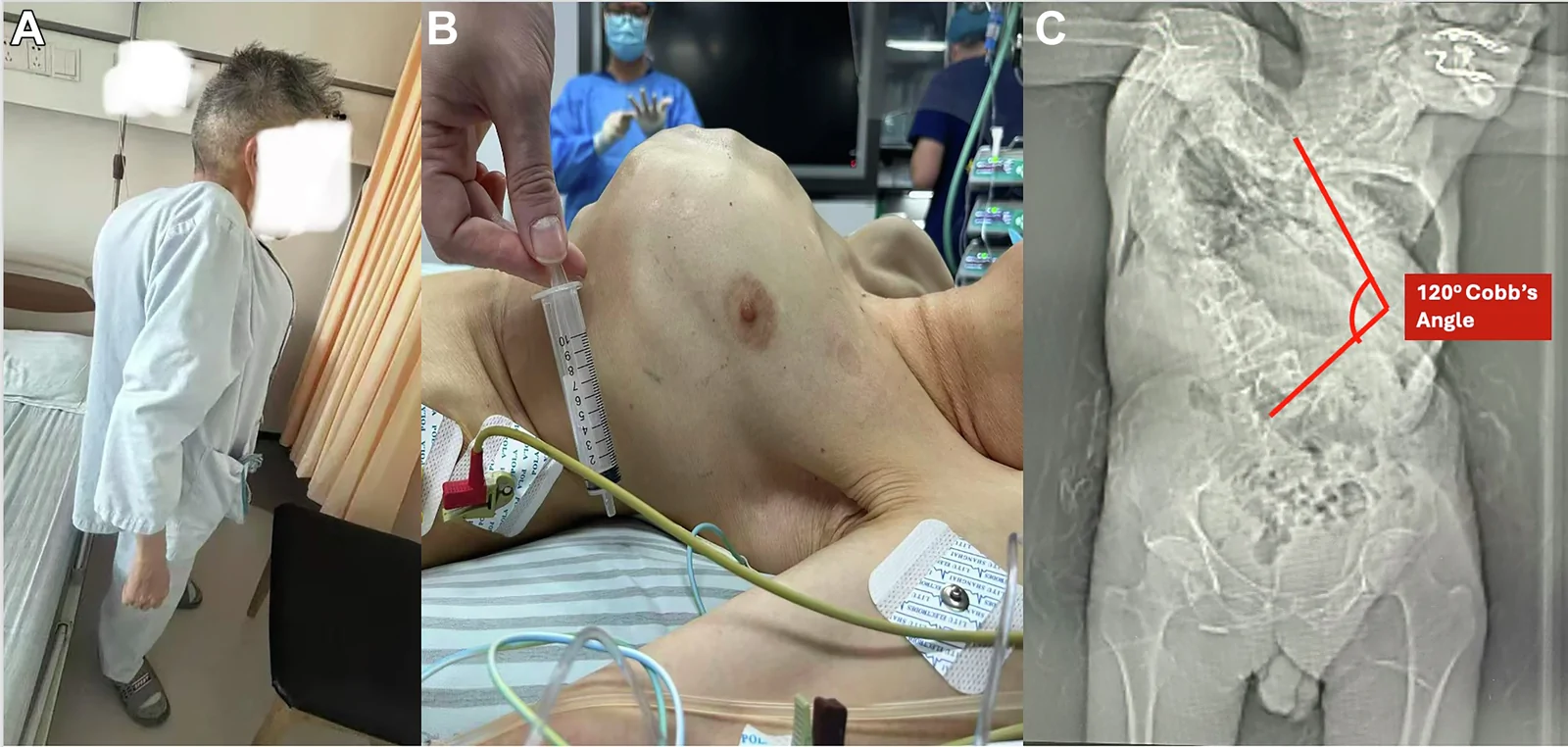
Postural Deformity of the Patient on Admission (A and B) Preoperative photograph of patient showing severe scoliotic curve. (C) Thoracic CT tomography shows a major thoracic curve of is 120 degrees Cobb angle. CT = computed tomography.

## Past Medical History

The patient had multiple comorbidities including being underweight (BMI of 16), hypertension, type 2 diabetes mellitus, stage III chronic kidney disease (estimated glomerular filtration rate 40 mL/min), chronic obstructive pulmonary disease, and severe scoliosis.

## Differential Diagnosis

The differential diagnosis included ruptured aortic sinus aneurysm and degenerative valvular heart disease which would be evident in echocardiography. Transesophageal echocardiography (TEE) was helpful for differential diagnosis. Considering the patient's medical history, along with the current physical examination and echocardiographic findings, there is a strong suggestion of CAMR.

## Investigations

TEE revealed right coronary aortic valve prolapse with severe AR and posterior mitral valve prolapse with severe MR. Dilated left ventricle (LV) with normal overall systolic function. The pertinent components of TEE are showed in Figure 2.

**Figure 2:**
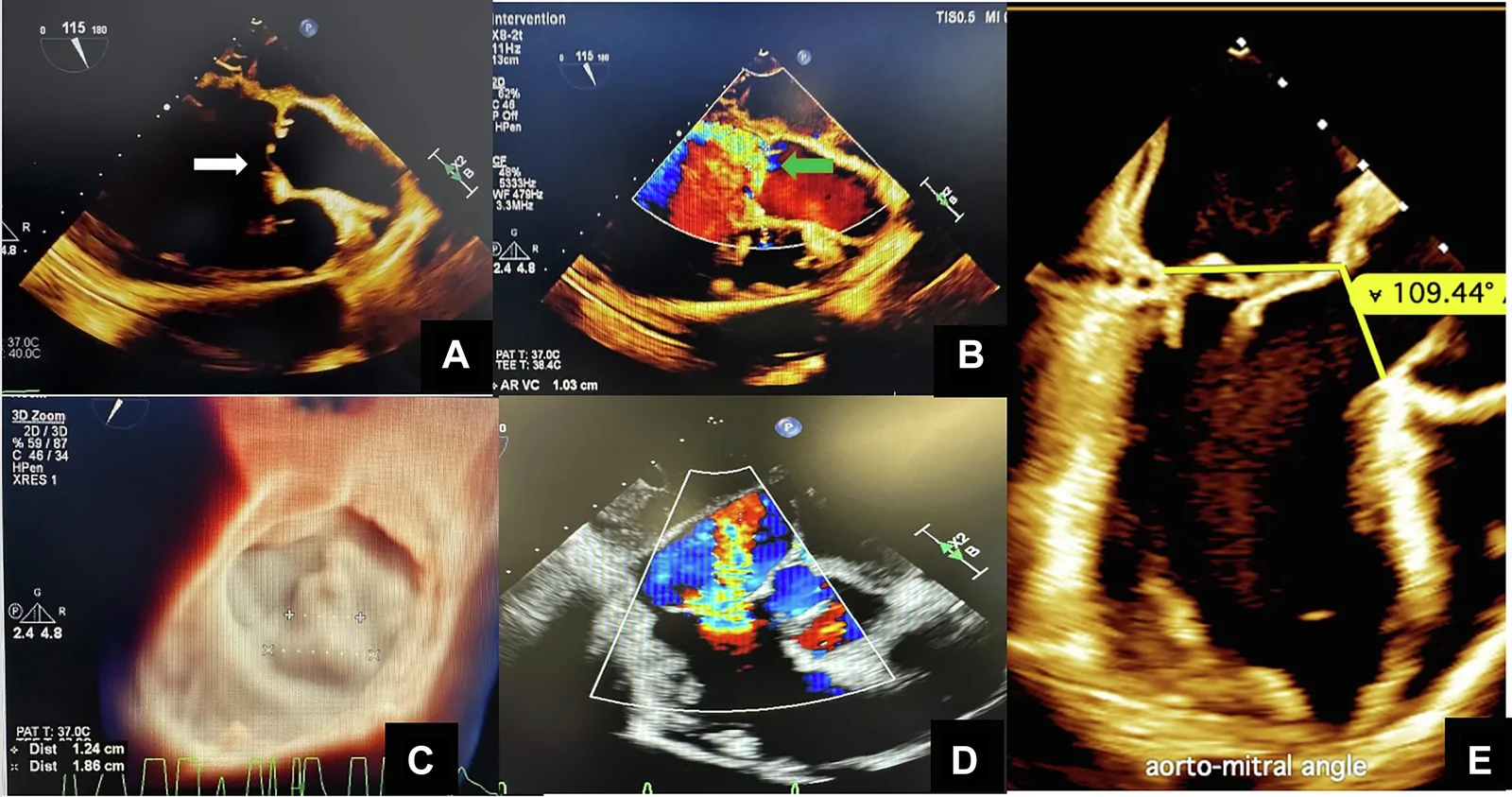
Evaluation of Aortic Regurgitation and Mitral Regurgitation by Transesophageal Echocardiography (A) Prolapse of the aortic right coronary cusps of the aortic valve seen on TEE (white arrow). (B) Eccentric jet aortic regurgitation visible on color Doppler; vena contracta = 10.3 mm (green arrow). (C) Three-dimensional en face surgeon's view shows P2 prolapse of the mitral valve with prolapse width of 18.6 mm. (D) Color doppler demonstrates mitral regurgitation PISA radius of 0.6 cm. (E) Aorto-mitral angle of 109° in TEE. 3D = three-dimensional; PISA = proximal isovelocity surface area; TEE = transesophageal echocardiography.

Cardiac computed tomography and computed tomography angiography showed extensive arterial tortuosity and severe scoliosis with no significant coronary artery disease (Figure 3). The pertinent components of pre-transcatheter aortic valve replacement (TAVR) report are showed in Figure 4.

**Figure 3:**
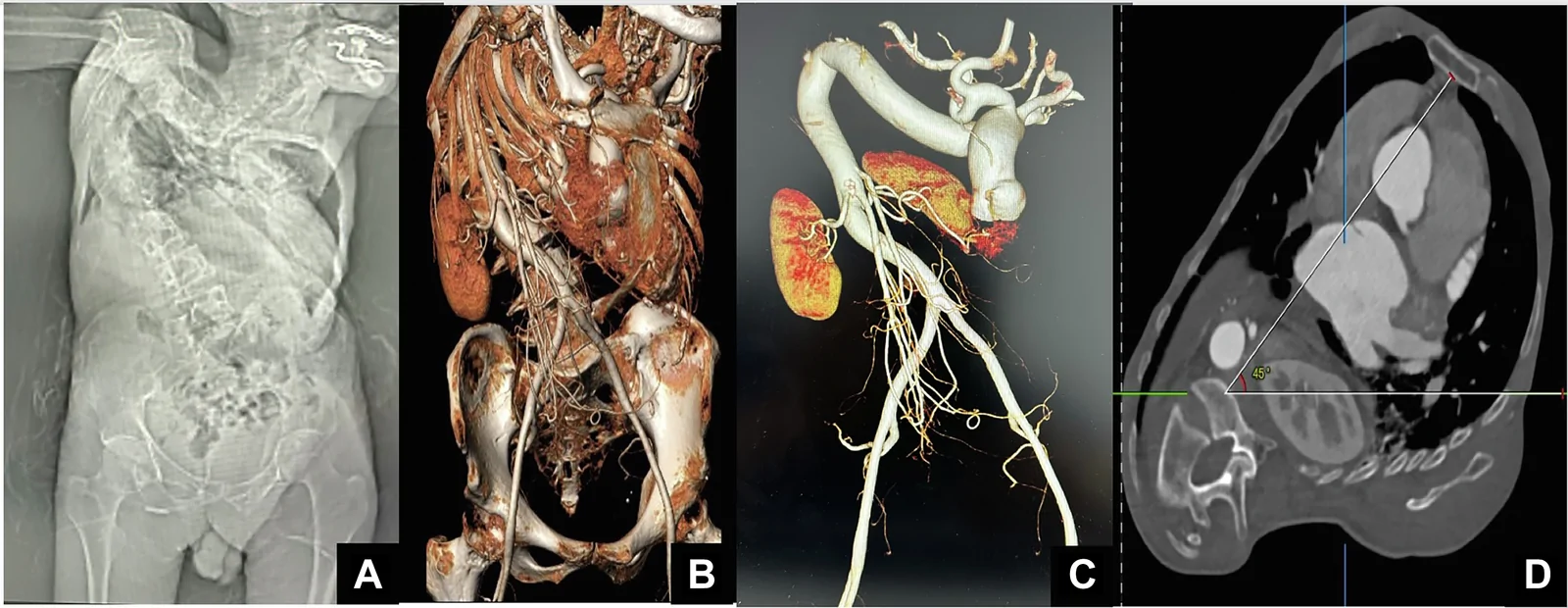
Evaluation of the Aorta by Computed Tomography (A) Preprocedural CT demonstrating severe scoliotic curve. (B) Three-dimensional volume-rendered reconstruction of the spine and the aorta in scoliosis patient with right curve. (C) Three-dimensional volume-rendered reconstruction of the aorta. (D) Axial CT showing aorto-vertebral angulation measurement of 45°. CT = computed tomography.

**Figure 4:**
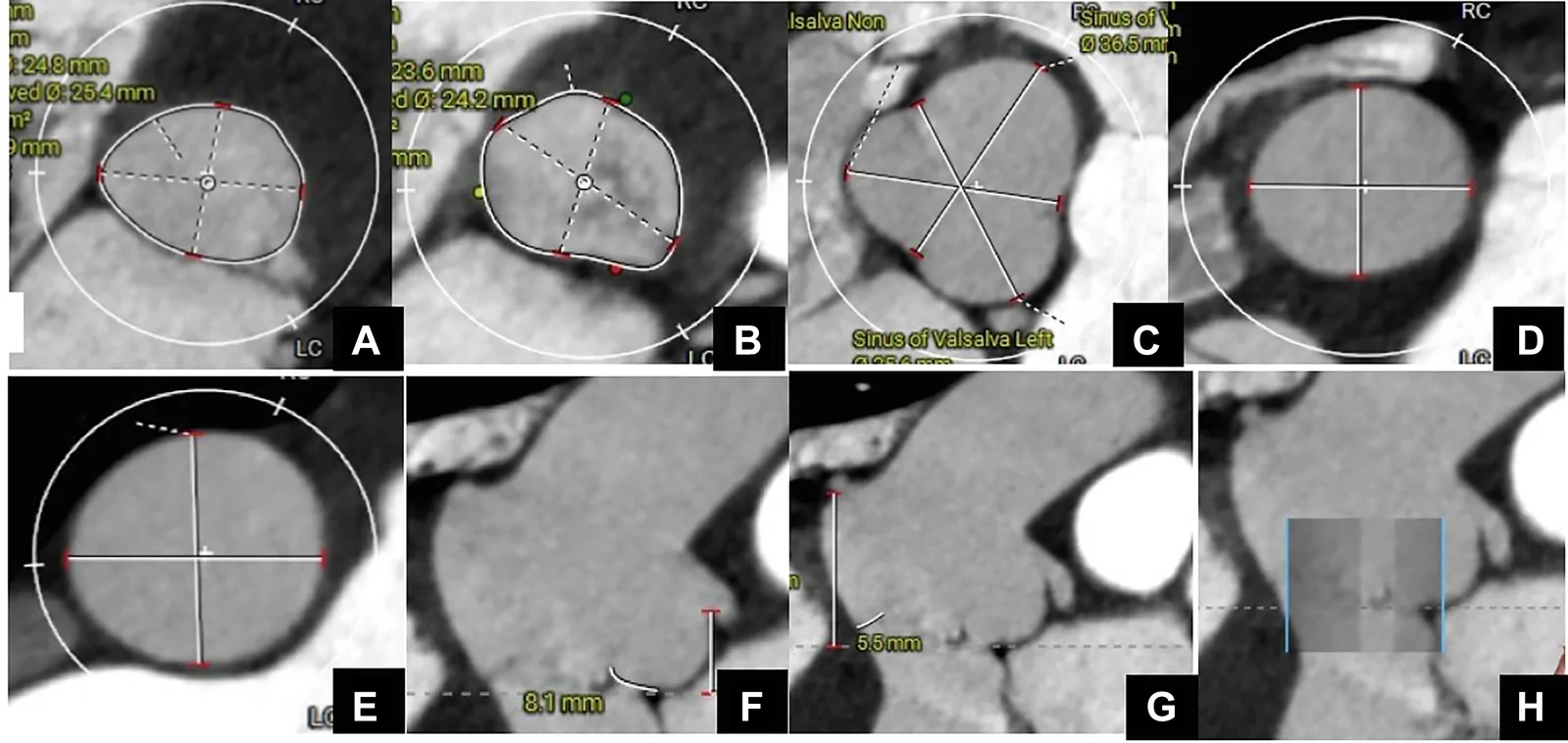
Evaluation of the Aortic Root and the Degree of Oversizing Rate by Computed Tomography Angiography (A) LVOT area was 24.8 mm. (B) Annular area was 23.6 mm. (C) SOV diameter was 35.6 mm, 36.5 mm, and 35.2 mm in the left, right, and noncoronary, respectively. (D) STJ diameter was 27.9 mm. (E) Ascending aorta diameter was 29.6 mm. (F) Left coronary height was 11 mm. (G) Right coronary height was 27 mm. (H) Computational modelling of 27-mm on-label SEV TAVR device in the aortic root. LVOT = left ventricular outflow tract; SEV = self-expanding valve; SOV = sinus of valsalva; STJ = sino-tubular junction; TAVR = transcatheter aortic valve replacement.

## Management

After admission, diuretic therapy was initiated for 7 consecutive days, which provided minimal relief of dyspnea. Given his high surgical risk (EUROScore II of 8.1%; STS PROM of 7.75%) for a double valve open heart surgery, a one-stage transapical mitral transcatheter edge-to-edge repair (mTEER) and TAVR were planned by the heart team.

## Surgical Access

In the hybrid catheterization laboratory, the heart team decided to perform an mTEER-first strategy. Transapical access was achieved by left anterolateral mini thoracotomy. Under TEE and fluoroscopy guidance, LV entry at the posterior-lateral apex provided adequate coaxial alignment for both TAVR and mTEER delivery (Figure 4). A 7F sheath was inserted at the center of the purse-string suture using a 0.35 J-wire, and a 6F JR4 catheter was advanced into the descending aorta.

## Transapical Mitral TEER Implantation for MR

The 7F sheath was replaced with a 16F sheath, and proper alignment towards the mitral valve was achieved under TEE guidance (Figures 4A and 4B). The mTEER device was positioned perpendicular to the mitral valve securing the A2-P2 mitral valve leaflets. The main steps of the device implantation are showed in Figure 5. There was resolution of A2-P2 regurgitation with a mean transmitral pressure gradient of 2.34 mm Hg after mTEER device implantation indicating procedural success.

**Figure 5:**
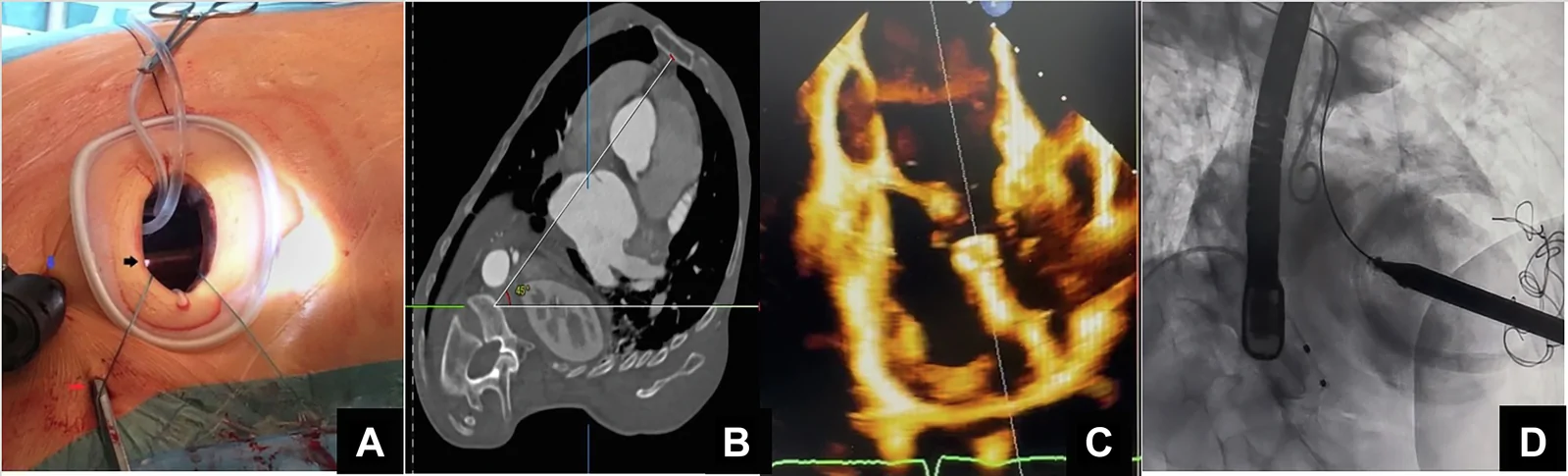
Access Site Selection for Mitral TEER and TAVR (A) Left antero-lateral mini thoracotomy. (B) Axial CT showing selected left ventricle puncture site (blue line). (C) TEE guided site selection for mitral TEER delivery system. (D) Fluoroscopy guided site selection for on-label SEV TAVR delivery system. CT = computed tomography; SEV = self-expanding valve; TAVR = transcatheter aortic valve replacement; TEE = transesophageal echocardiography; TEER = transcatheter edge-to-edge repair.

## Transapical On-Label TAVR Implantation for AR

Subsequently, the mTEER system was replaced with 24F dilator to dilate the puncture site at the LV apex. Using 0.35 stiff wire, a 27-mm on-label TAVR device was deployed in a two-step process, and the anchor rings were deployed in the aortic sinuses followed by the aortic valve prosthesis in the aortic annulus. The main steps of the device implantation are showed in Figure 6. Achieving coaxial alignment during deployment was challenging due to a highly horizontal aorta (aortic-ventricular angle of 125°) (Figure 7F). Following TAVR, there were no significant aortic and paravalvular regurgitations indicating procedural success. The valve system was withdrawn, and the purse-string suture was subsequently tied.

**Figure 6:**
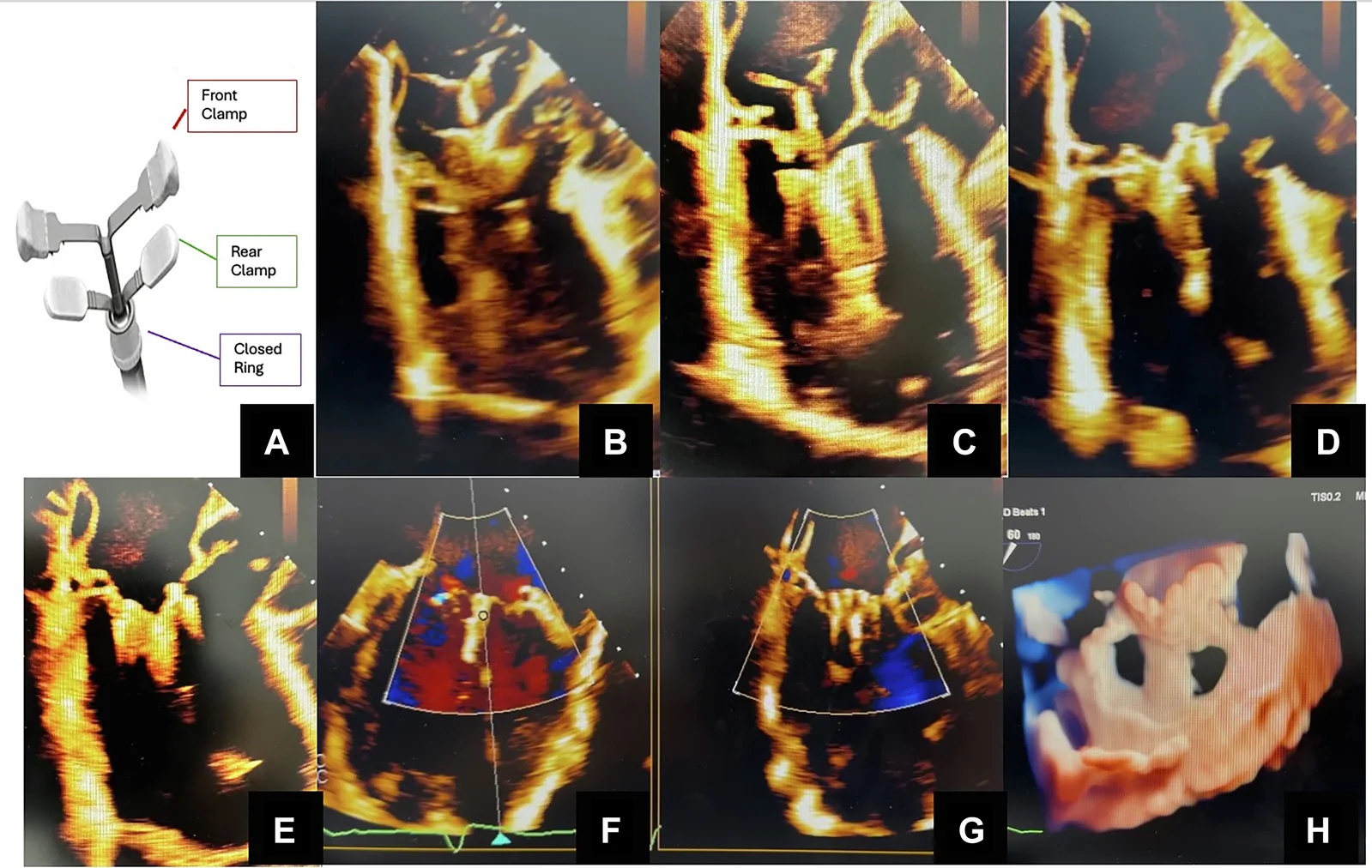
The Main Steps of Transapical Mitral TEER Implantation for Mitral Regurgitation (A) Mitral TEER device composed of front clamp, rear clamp, and closed ring. (B) The clamp is delivered and opened within the left atrium. (C) The rear clamp is placed under the leaflets, and the front clamp remains in the left atrium. (D) The front clamp is pulled back to capture the leaflets, then the closed ring is moved forward to cover the ventricular end of the clamp arms. (E) The clamp is released. (F and G) Color doppler showed insignificant mitral regurgitation after mitral TEER. (H) Three-dimensional reconstruction demonstrated mitral valve dual orifice areas after mitral TEER. TEER = transcatheter edge-to-edge repair.

**Figure 7:**
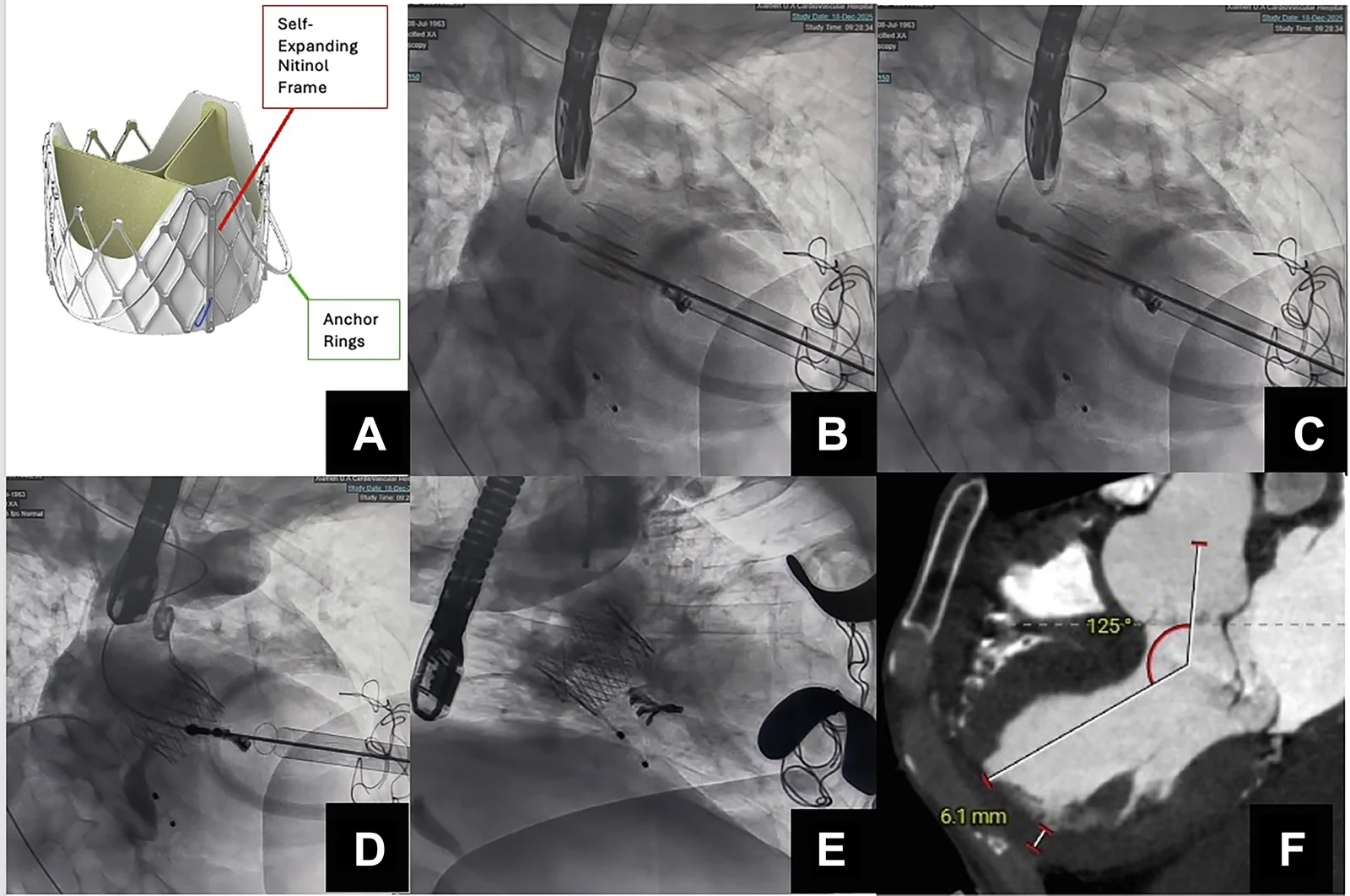
The Main Steps of On-label TAVR Implantation for Aortic Regurgitation (A) The 27-mm transapical on-label SEV TAVR device for aortic regurgitation consisting of bovine pericardial leaflets within a nitinol stent frame deployed within a nitinol anchor ring. (B) Prosthetic valve was inserted above the aortic annulus. (C) Anchor rings were placed in aortic sinuses respectively. (D) Prosthetic valve was pulled back to the aortic annulus. (E) Prosthetic valve was released, and native aortic valve was fixed between prosthesis and locators. (F) CT showing LV long axis and the line that passes through the center of the aorta (aortic-ventricular of 125°). LV = left ventricle; SEV = self-expanding valve; TAVR = transcatheter aortic valve replacement.

## Outcome

There was no significant blood loss which required transfusion, and the total procedure time was 120 minutes. TEE following mTEER demonstrated stable positioning of the mTEER device, resulting in a double orifice mitral valve configuration with a maximum transmitral gradient of 5 mm Hg and no significant MR (Figures 7A to 7C).

Post-TAVR TEE confirmed normal bioprosthetic aortic valve motion with only trivial paravalvular regurgitation, a peak aortic valve velocity of 1.8 m/s, and a transaortic gradient of 6 mm Hg (Figures 7D to 7F). The patient was transferred to the intensive care unit without a need for inotropic support. Noninvasive hemodynamic monitoring demonstrated preserved global cardiac function (cardiac output of 4.1 L/min and a cardiac index of 3.9 L/min/m^2^).

At 30-day follow-up, transthoracic echocardiography demonstrated a well-functioning aortic bioprosthesis (effective orifice area of 1.8 cm^2^, peak gradient of 16 mm Hg, and mean gradient 8 mm Hg) with no significant regurgitation. The mitral valve clamp remained well seated, with a maximal transmitral gradient of 6 mm Hg, a mean gradient of 2 mm Hg, and no significant regurgitation. Overall LV systolic function was preserved (LV ejection fraction of 57%).

## Discussion

CAMR in scoliosis is a rare valvulopathy with a reported prevalence of 0.35%.^1^ When both valves exhibit severe regurgitation simultaneously, it leads to impaired cardiac function, heightened operative risk, and poorer prognosis.^2^ Evidence guiding the management of severe CAMR remains limited.

In cases of severe AR and MR, treatment of both valves is warranted. Surgical mitral intervention is class I recommendation for primary MR with aortic valve replacement^3^ and has been shown to be survival-protective but is associated with a higher risk of postoperative LV dysfunction.^2^ The current guideline has emphasized transcatheter treatment of a single dominant lesion of multivalvular disease. Most studies reported increased risk of mortality when coexistent significant MR was present at the time of TAVR.^3^

A review of 37 studies involving 60 high-risk patients undergoing combined transcatheter aortic and mitral intervention found that only 5 had combined AR and MR with a 15% mortality rate in 419 days conferring reasonable clinical outcome.^4^

The critical consideration in this case was the selection between one-stage vs two-stage treatment strategy for CAMR. A two-staged approach entails addressing the aortic valve first, with subsequent mitral valve intervention for patients who remain symptomatic^3^ because improvement in MR severity following TAVR has been reported, especially MR of functional etiology while MR may remain unchanged after successful TAVR in the presence of degenerated and calcified mitral valve disease.^5^

In contrast, a one-stage approach involves simultaneous treatment of both lesions to avoid treatment delays.^3^ Moreover, there was no difference in in-hospital mortality between one-stage vs a two-stage approach.^6^

Another key consideration was the selection of access route. Although the transvessel approach for transcatheter intervention is associated with lower morbidity and mortality, in this case, marked distortion of the thoracoabdominal aortic course was observed, producing an unfavorable aorto-vertebral angulation and challenging device coaxiality for TAVR and mTEER.

TA access allows direct LV access and facilitates precise device deployment.^7^ In this case, a left anterolateral mini thoracotomy with LV entry at the posterior-lateral apex was performed. Although posterior-lateral access (Figure 3D) is not favored for TA TAVR, it did not preclude achievement of adequate coaxiality during deployment.

An additional caveat of TA access, particularly in elderly patients, is the risk of apical adhesions and localized myocardial weakness, which may increase the likelihood of LV rupture.^8^ This risk may be compounded in a two-stage approach favoring a one-stage approach.

The use of a dedicated device is another key factor for procedural success. In the present case, mitral valve prolapse was successfully repaired using a single mTEER device with clamp arms, which permits complete opening of the arms allowing a larger capture range and precise control compared to the conventional mTEER device (Figure 6A). Notably, CLAMP-2 trial reported an 87% 1-year primary efficacy endpoint and an all-cause mortality of 3.9%.^9^

The absence of leaflet calcification in native AR poses significant challenges for valve anchoring during TAVR when off-label devices are used.^10^ In contrast, on-label TAVR device incorporates 3 U-shaped device graspers that provide extra-axial fixation, making it suitable for the present case (Figure 7A). Despite improved transcatheter heart valve platform and techniques, TAVR for pure severe native aortic valve remains a challenging procedure, with significant risk for transcatheter valve embolization; hence, TAVR maybe considered an effective therapeutic option when performed with a dedicated device to those at high risk who are unsuitable candidates for surgery.^10^

The procedure sequence was also critical to procedural success in this case. The optimal procedural sequence for concomitant TA-TAVR and TA-mTEER remains undefined. In the present case, the decision to perform a transapical mTEER-first strategy (Figure 8) was guided by both hemodynamic and technical considerations. Reduction of MR before TAVR may improve left atrial pressure and pulmonary venous congestion, potentially facilitating subsequent aortic intervention.

**Figure 8:**
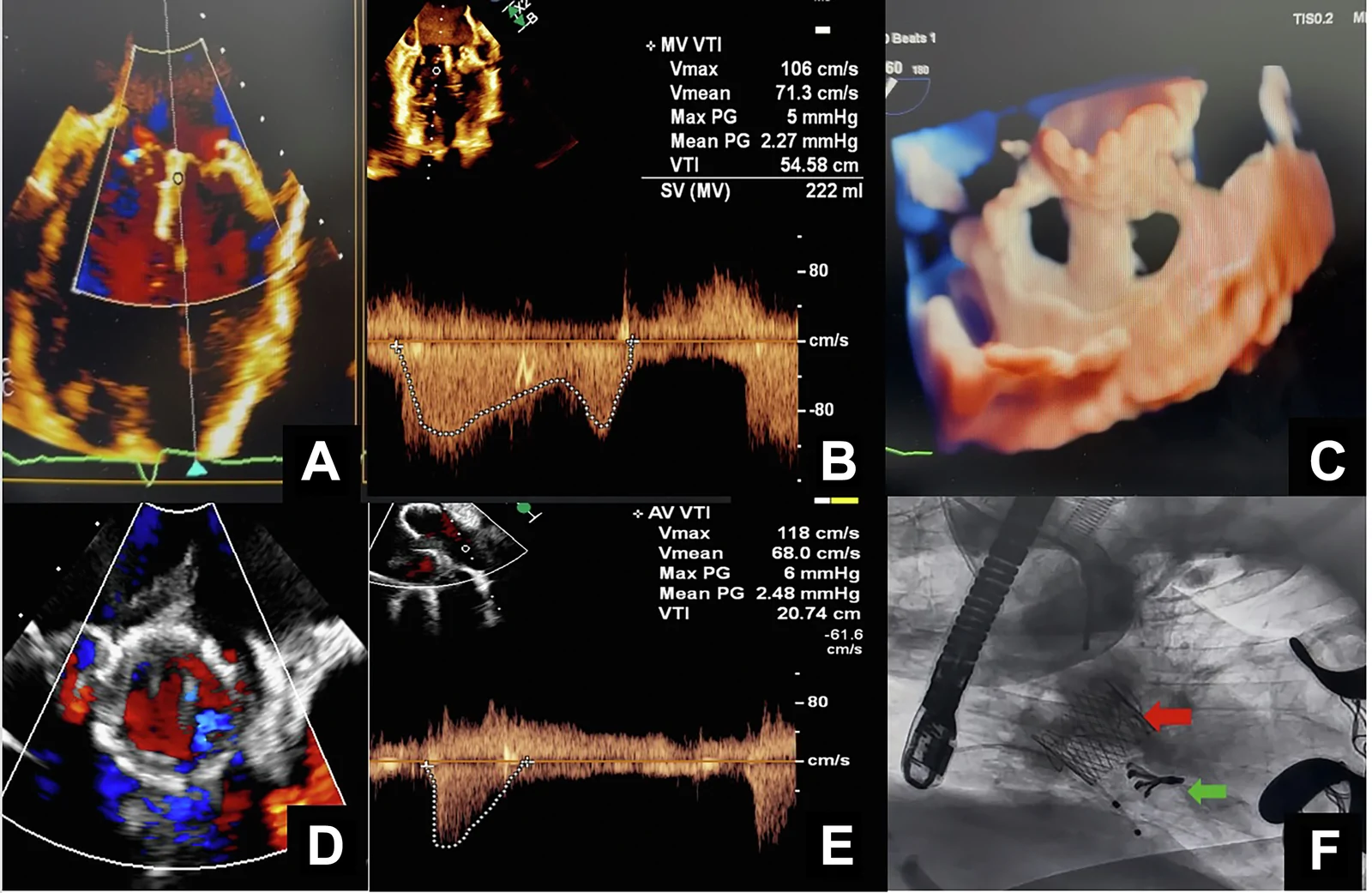
Final Result After Transapical Mitral TEER and Transapical TAVR (A) Two-dimensional TEE and color doppler showing mTEER device in good position and stable with no significant regurgitation. (B) Transmitral doppler derived maximum gradient is 5 mm Hg. (C) Three-dimensional TEE en face view of mitral valve dual orifice. (D) Color doppler showed no significant paravalvular leakage after TAVR. (E) Transaortic doppler derived gradient is 6 mm Hg. (F) Fluoroscopy demonstrates good and stable position of the on-label TAVR (red arrow) and mTEER device (green arrow) with no significant regurgitation and paravalvular leak. mTEER = mitral transcatheter edge-to-edge repair; TAVR = transcatheter aortic valve replacement; TEE = transesophageal echocardiography.

The aorto-mitral angle in this case is 109°, indicating an acute mitral-aortic coupling with compact left ventricular outflow tract geometry with a higher likelihood of device interaction. A transapical TAVR-first strategy will cause the anterior mitral leaflet to move closer to the aortic prosthesis, changes in the LV geometry, and sudden drop of the LV afterload leading to a challenging mTEER imaging, trajectory toward A2-P2, leaflet grasp optimization, and capture maneuverability. TAVR implantation depth <4 mm has been reported to reduce the risk of such interference.^4^

Conversely, a transapical TAVR-first strategy offers immediate correction of the dominant left ventricular volume overload imposed by severe AR, which improves effective forward stroke volume, and changes in ventricular dimensions and leaflet coaptation following restoration of forward flow can influence clip strategy. From a technical standpoint, performing a TAVR-first strategy may minimize the risk of interaction between large-bore transapical delivery systems and a previously repaired mitral valve.

In the absence of established guidelines, procedural sequencing should therefore be individualized according to valve pathology dominance, hemodynamic status, and technical anatomy. As with other combined valve diseases, multidisciplinary decisions were made to better define optimal patient selection, procedural sequencing, and access strategies to optimize outcomes.

**Visual Summary:**
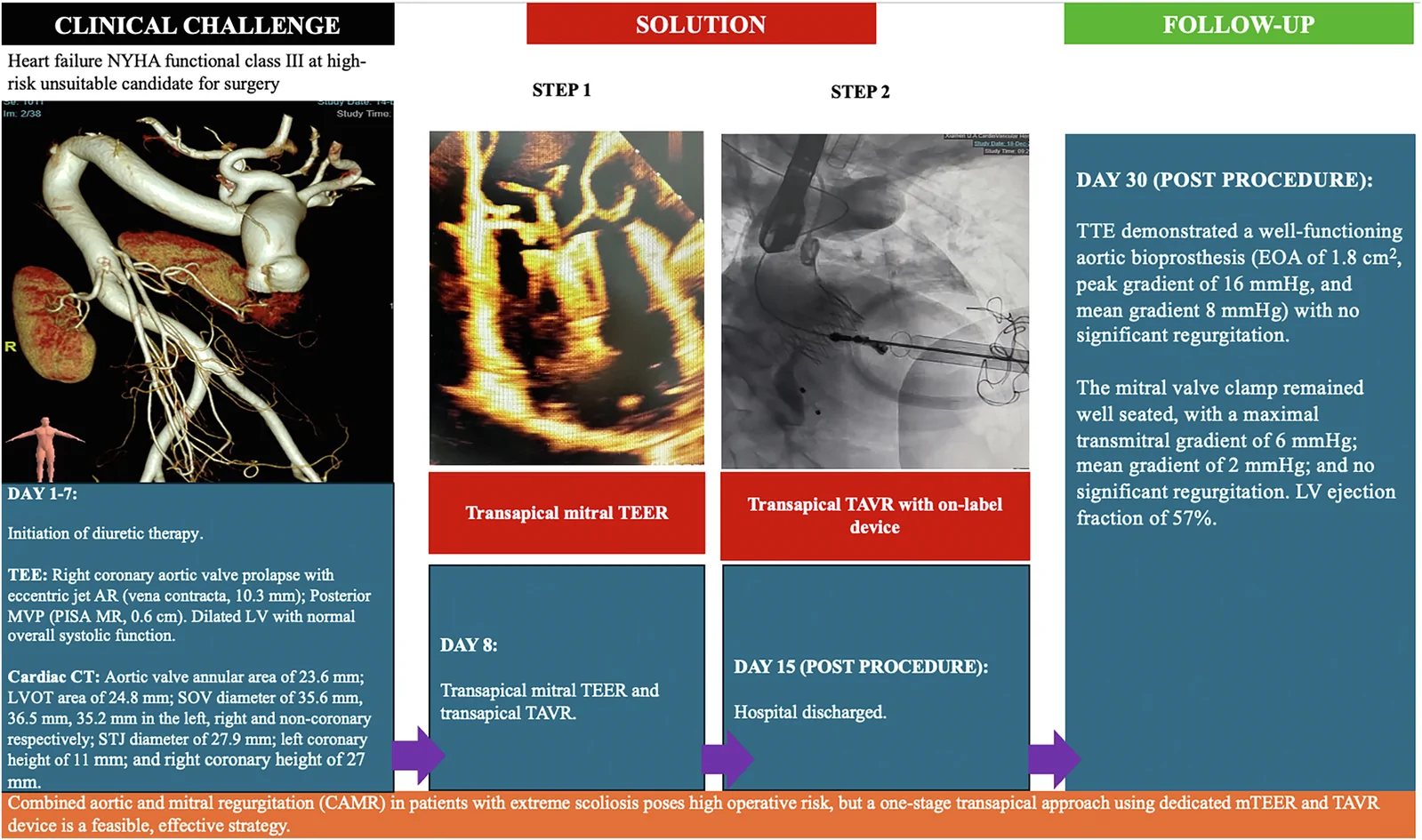
One-Staged Transcatheter Intervention for CAMR in Patient With Extreme Scoliosis AR = aortic regurgitation; CT = computed tomography; CTA = computed tomography aortogram; EOA = effective orifice area; LVEDD = left ventricular end diastolic diameter; LVEF = left ventricular ejection fraction; LVOT = left ventricular outflow tract; MR = mitral regurgitation; MVP = mitral valve prolapse; NYHA = New York Heart Association; PISA = proximal isovelocity surface area; SOV = sinus of valsalva; STJ = sinotubular junction; TAVR = transcatheter aortic valve replacement; TEE = transesophageal echocardiography; TEER = transcatheter edge-to-edge repair; TTE = transthoracic echocardiography.

**Equipment List:** List of Equipment Used in This Case

Transapical mitral transcatheter edge-to-edge repair	
Imaging	TEE machine (Philips Healthcare) 0.035-inch X7 TEE probe
Access	TEE machine (Philips Healthcare) Micropuncture needle and wire 0.035-inch J wire and 7F sheath 0.035-inch Amplatz super stiff guidewire (Boston Scientific) 16F sheath (APT Medical)
Mitral TEER device	Mitral ValveClamp and delivery system (Shanghai Hanyu Medical)
Transapical transcatheter aortic valve replacement	
Imaging	TEE machine (Philips Healthcare) 0.035-inch X7 TEE probe
Access	TEE machine (Philips Healthcare) 24F dilator 0.035-inch J wire Lunderquist extra stiff guidewire (Cook)
TAVR device	27-mm J-Valve and delivery system (JieCheng Medical)

TAVR = transcatheter aortic valve replacement; TEE = transesophageal echocardiography; TEER = transcatheter edge-to-edge repair.

## Funding Support and Author Disclosures

The authors have reported that they have no relationships relevant to the contents of this paper to disclose.
